# Association of macrophage inhibitory factor -1 polymorphisms with antiviral efficacy of type 1b chronic hepatitis C

**DOI:** 10.1007/s11010-021-04097-2

**Published:** 2021-02-18

**Authors:** Songdao Ye, Yao Chen, Xiaoting Lou, Xuanmei Ye, Xunjun Yang

**Affiliations:** 1 Department of Laboratory Medicine, The Second Affiliated Hospital and Yuying Children’s Hospital of Wenzhou Medical University, Wenzhou, Zhejiang China; 2 Key Laboratory of Laboratory Medicine, Ministry of Education, Zhejiang Provincial Key Laboratory of Medical Genetics, Wenzhou Medical University, Wenzhou, Zhejiang China; 3 Department of Pathology, The First Affiliated Hospital of Wenzhou Medical University, Wenzhou, Zhejiang China

**Keywords:** Macrophage inhibitory factor-1, Chronic hepatitis C, Single nucleotide polymorphisms, Antiviral therapy

## Abstract

The expression of macrophage inhibitory factor-1 (MIC-1) increases in patients with chronic hepatitis C (CHC), but whether MIC-1 level and its polymorphism affect the antiviral efficacy of CHC has not yet been reported. The present study aimed to investigate the association between MIC-1 polymorphism and antiviral efficacy in patients with CHC genotype 1b (CHC 1b). A total of 171 patients with CHC1b were recruited. The polymorphisms of rs1059369 and rs1059519 in *MIC-1* were detected by DNA sequencing. All patients received a standard dose of polyethylene glycol interferon + ribavirin (PR regimen), and divided into response, nonresponse, sustained virological response (SVR), and non-sustained virological response (NSVR) groups based on HCV RNA levels. The genotype distribution of the two single nucleotide polymorphisms (SNPs) did not differ between the response and nonresponse groups, SVR and non-SVR groups. However, the level of MIC-1 was positively correlated with ALT, AST, PIIINP, CIV, and *HCV* RNA (*P* < 0.05). Compared to before treatment, the level of MIC-1 in plasma was significantly decrease in the response group but not in the non-responsive group. Our results suggest that the level of MIC-1 in CHC1b is correlated with liver cell injury, liver fibrosis index, and viral load. However, the polymorphism of rs1059369 and rs1059519 may have negligible impact in expression of MIC-1 and efficacy of antiviral therapy in CHC patient.

## Introduction

Hepatitis C is a progressive disease characterized by the extrahepatic disease, which is caused by the hepatitis C virus (HCV) infection. It has affected more than 185 million people worldwide. The disease develops into liver fibrosis, liver cirrhosis, and hepatocellular carcinoma due to lack of an effective vaccine [1, 2]. HCV infection causes about 350,000 deaths each year, and hence, the mechanism underlying chronic hepatitis C (CHC) and how to predict and improve the response rate of treatment are under intensive research [3, 4]. Direct-acting antiviral agents (DAA) drugs have been approved for marketing in China. Although these drugs have several advantages, they are still in the initial stage of clinical application. Also, since these drugs are not cost-effective, polyethylene glycol interferon + ribavirin (PR regimen) is the primary treatment of hepatitis C in China. Currently, the *HCV* gene is easily variable and can be divided into at least six genotypes and several subtypes. In China, 1b genotype and 2a genotype are the main types, and SVR was obtained in 50–70% of the patients with genotype 1 after 48 weeks of standard therapy [5]. Factors related to efficacy, included viral load, viral genotype [6, 7], and host factors (such as age, sex, SNP, and immune status) [8].

MIC-1, also known as growth differentiation factor-15 (GDF-15), is a member of the transforming growth factor-beta (TGF-β) superfamily. MIC-1 is downregulated in healthy individuals; however, the expression was upregulated under the pathological or stress effects, such as inflammation and trauma [9]. MIC-1 is involved in multiple organ injury and disease progression via the modulation of the inflammatory response and apoptosis pathways, such as cardiovascular disease [10–12], malignant tumor [13–15], and diabetes [16]. In recent years, MIC-1 expression has been found to be elevated in patients with chronic hepatitis [17], cirrhosis [18], and small cell carcinoma of the liver [19]. MIC-1 is valuable in the diagnosis and prediction of chronic hepatitis complications [20]. There are many SNPs in the human *MIC-1* gene, and most of them are nonsense SNPs, while SNPs in exons may cause changes in the amino acid sequence of the MIC-1 precursor protein. Among them, rsl059519 and 1059369 are the most focused sites, several studies have found that the polymorphisms of MIC-1 are crucial genetic factors on cardiovascular diseases [21, 22] and malignant tumors [23]. However, There are no report about influence of MIC-1 gene polymorphism in hepatitis b virus so far. Thus, the present study aimed to assess the correlation between the level of MIC-1 and rs1059519 and rs1059369 polymorphisms in the exon region and the efficacy of PR regimen in patients with CHC.

## Materials and methods

### Study population

A total of 171 CHC patients were recruited in this study from the Infection Department of the Second Affiliated Hospital of Wenzhou Medical College between September 2016 and August 2019. The diagnostic criteria of “Hepatitis C prevention and treatment guidelines” put forth in 2015 were followed. The inclusion criteria were as follows: age 18–70 years, HCV infection for > 6 months or epidemiological history of 6 months, positive for anti-HCV and *HCV* RNA, histopathology of liver, HCV1b, no antiviral treatment and use of immunomodulators within 3 months, no absolute contraindication of PR treatment, can receive PR treatment, and signed the informed consent. The exclusion criteria were as follows: a combination of chronic hepatitis B and other hepatitis, alcoholic liver disease, drug-induced liver injury, autoimmune hepatitis, and other liver diseases, HIV infection, malignant tumors, patients with severe cerebrovascular disease, hematological disease, thyroid disease, diabetes, and incomplete data, which might affect efficacy and safety. This study was approved by the Medical Ethics Committee of the Second Affiliated Hospital of Wenzhou Medical University, which conformed to the ethical guidelines of the Helsinki Declaration. Informed consent was obtained from the enrolled patients.

To evaluate the efficacy, all patients were treated with PR therapy. Usage: IFN-2A (Shanghai Roche Pharmaceutical, J20120074) subcutaneous injection, 180 μg once per week; oral administration of 800–1000 mg/d ribavirin (Shanghai Xinyi Tianping Pharmaceutical, H10960157). The basic course of treatment was 48 weeks. *HCV* RNA was monitored before treatment and at 4, 12, 24, and 48 weeks’ post-treatment.

Efficacy evaluation and grouping [4]: According to the level of *HCV* RNA at the 12th week of treatment, the patients were divided into two groups: (1) virological response: *HCV* RNA decreased ≥ 2 log at the 12th week of treatment as compared to pre-treatment; (2) no virological response: *HCV* RNA decreased 2 log at the 12th week of treatment as compared to pre-treatment. According to the level of *HCV* RNA at the 24th week of follow-up post-treatment, *HCV* RNA was classified into sustained virological response (SVR): *HCV* RNA at the 24th week after treatment could not be measured and non-SVR group: *HCV* RNA was still detectable at 24 weeks after the treatment.

### Blood samples

A volume of 3–4 mL venous blood was collected on an empty stomach and mixed in the EDTA-K2 anticoagulant tube. Blood routine was performed by counting platelets (PLT). The plasma and blood cells were separated by centrifugation at 3000 rpm for 5 min and stored at − 80 °C. The plasma was used to detect level of ALT, AST, TP, Alb, MIC-1, *HCV* RNA, and blood cells were used for DNA extraction and polymorphism analysis. Venous blood was collected again at the end of the basic course of treatment (48 weeks), and plasma was collected and stored to detect the level of MIC-1.

### DNA extraction and PCR amplification

Genomic DNA was extracted and amplified using the blood genomic DNA extraction kit (Shenggong Biological Engineering, Shanghai, China, SK8224), according to the manufacturer’s instructions. DNA was amplified using a PCR kit (Shenggong Biological Engineering, Shanghai, China, SK2072) and Verity 96-well PCR instrument (Applied Biosys-tems, Foster City, CA, USA). The primers were synthesized by Shanghai Bioengineering, rs1059369-rs1059519-F: 5′tacctctctctggctgagtccg3′, rs1059369-rs1059519-R: 5′gcggagaccaaagt3′. The PCR amplification was as follows: pre-denaturation at 95 °C for 3 min, 35 cycles of denaturation at 94 °C for 30 s, annealing at 58 °C for 30 s, and extension at 72 °C for 50 s, repair and extension at 72 °C for 10 min, and preservation at 4 °C.

### Purification and sequencing of PCR products

The amplified products were purified by PCR product purification kit (Shenggong Biological Engineering, Shanghai, China, SK1141) and estimated on Nanodrop 2000C UV photometer (Thermo, USA). The extracted DNA was sequenced on a 3730XL sequencing instrument (Applied Biosystems, Foster City, CA, USA) and analyzed using SeqMan software for a comparative analysis of the sequence map [24].

### Quantitation of the level of MIC-1

The level of MIC-1 in plasma was determined by the double antibody sandwich enzyme-linked immunosorbent assay (ELISA), according to the manufacturer’s instructions (Sujingmei Biotechnology, Shanghai, China). The OD values of the samples and standard samples were detected by Anthos 2010 enzyme-labeled instrument (Shanghai Bosses Technology, China), and the level of MIC-1 was extrapolated on the standard curve.

### Quantitation of HCV RNA

HCV RNA ≥ 5.0102 copies/mL was the abnormality as detected by real-time fluorescent quantitative polymerase chain reaction (qRT-PCR), according to the manufacturer’s instructions (Piji Bioengineering, Shenzhen, China) on the ABI7500 Fluorescent Quantitative PCR Instrument (Applied Biosystems, USA) [25].

### Quantitation of plasma biochemical indexes

ALT, AST, TP, ALB, and TBIL levels in plasma were measured by ADVIA2400 Automatic Biochemical Analyzer (Siemens, Berlin, GER). The normal reference range was as follows: ALT: 9–50/L, AST: 15–40/L, TP: 65–85 g/L, ALB: 40–55 g/L, TBIL: 6.8–34.2 μmol/L.

The levels of N-terminal peptide of type III procollagen (PIIINP) and type IV collagen (CIV) were determined by chemiluminescent immunoassay (CLIA) on a Maglumi 2000 Automatic CLIA (Shenzhen New Industry Biology); the normal reference range of PIIINP was 0.5–30 ng/mL and that of CIV was 5.0–30 ng/mL.

### Statistical analysis

All statistical analyses were performed using SPSS23.0. Pearson’s chi-square test or Fisher’s exact probability test was used for comparing the enumeration data between groups, mean and standard deviation was used to describe the normal distribution data, and *t*-test or variance analysis was used to compare the count data between groups. Median (M), 5th percentile (P5), and 95th percentile (P95) were used to represent the non-normal distribution data, and the rank sum test was used for the inter-group comparison. Significant differences were noted in the genotype and allele frequency. Hardy–Weinberg balance test, odds ratio (OR), and 95% confidence interval (95% CI), linkage disequilibrium analysis, and haplotype construction were conducted using Shesis (http://analysis.biox.cn/myanalysis.php). All tests were bilateral, and *P*-value < 0.05 was considered statistically significant.

## Results

### Baseline characteristics

A total of 171 patients with type 1b CHC (CHC1b), consisted of 95 males (age 41.65 ± 10.42, range 18–70 years). The plasma ALT, AST, TP, Alb, TBIL, PIIINP, CIV, PLT, MIC-1, and other parameters were non-normally distributed and expressed as median (m), 5th percentile (P5), and 95th percentile (P95); ALT, PIIINP, CIV, and LG (*HCV* RNA) was significantly lower in the response group than the nonresponse group (*P* < 0.05). The levels of AST, TP, Alb, TBIL, PLT, MIC-1 were not significantly different from those in the non-responsive group. A significant difference was observed in the age distribution between the two groups (*P* < 0.05), but no difference was detected in the ratio of the gender (*P* > 0.05). The results are summarized in Table 1.

**Table 1:** Comparison of gender, age, and baseline characteristics between CHC 1b treatment response group and non-response group

Characteristics	Response group (*n* = 124)	Nonresponse group (*n *= 47)	Statistical value (χ^2^, Z)	*P*
Sex (Male/Female)	69/55	27/20	χ^2^ = 0.045	0.832
Age (years, ≤ 40/ > 40)	63/61	15/32	χ^2^ = 4.903	**0.027**
ALT(U/L, M/P5/P95)^a^	40.4/14.5/126.0	56.0/14.7/130.1	Z = − 2.107	**0.035**
AST(U/L, M/P5/P95)	36.9/18.6/104.3	45.8/17.8/119.4	Z = − 1.782	0.061
TP (g/L, M/P5/P95)^a^	73.1/54.7/85.1	68.8/53.5/85.4	Z = − 1.175	0.240
ALB (g/L, M/P5/P95)^a^	27.2/38.3/55.3	27.9/27.5/55.2	Z = − 0.794	0.427
TBIL (µmol/L, M/P5/P95)^a^	16.9/11.3/41.5	19.3/11.9/41.2	Z = − 1.886	0.059
PIIINP (ng/mL, M/P5/P95)^a^	27.3/10.1/115.4	31.7/10.3.6/151.2	Z = − 2.244	**0.025**
CIV (ng/mL, M/P5/P95)^a^	28.4/11.5/108.6	33.7/12.1/165.1	Z = − 2.325	**0.021**
PLT(10^9^/L, M/P5/P95)^a^	185.0/58.3/285.8	172.0/61.2/307.6	Z = − 0.426	0.670
MIC-1 (pg/mL, M/P5, P95)^a^	365.1/156.2/1540.3	644.4/146.6/1500.7	Z = − 1.699	0.089
Lg (HCV RNA)	6.57/2.94/7.39	6.89/5.27/7.66	Z = 2.225	**0.026**

Bold indicates a statistically significant difference (*P* < 0.05)

^a^Median (M), 5th percentile (P5), and 95th percentile (P95) were used to represent the non-normal distribution data

### Changes in plasma MIC-1 before and after treatment

**Fig. 1:**
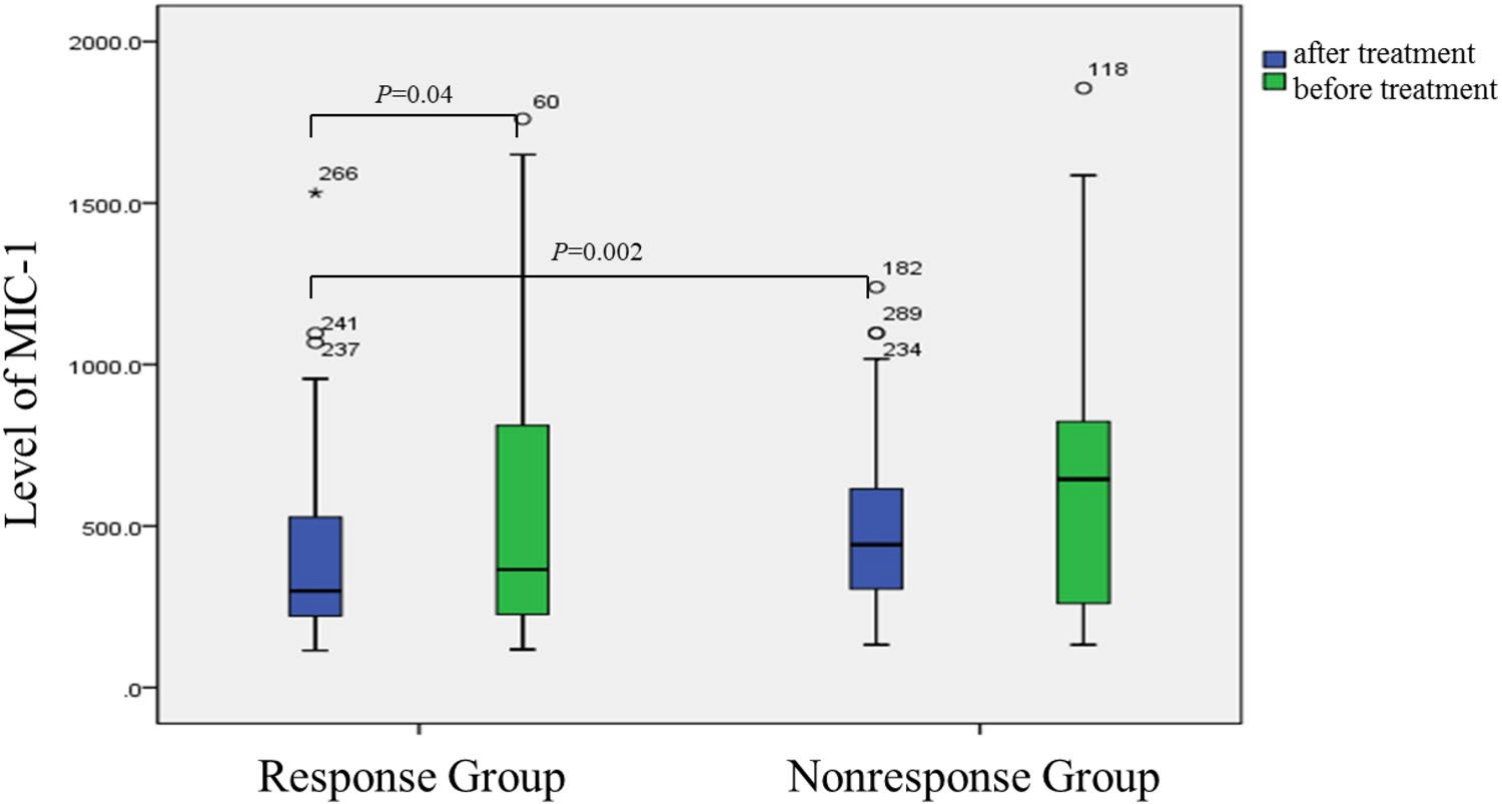
Comparison of CHC 1b patients before and after treatment of plasma MIC-1. The level of MIC-1 in the response group was significantly different between after treatment and before treatment (*P* = 0.04). Similarly, significant difference was observed in the MIC-1 between the response and nonresponse groups after treatment (*P* = 0.002)

After treatment, the level of MIC-1 (M-298.8 pg/mL, P5 = 159.9 pg/mL, P9 = 5952.7 pg/mL) in the response group was significantly lower than that before treatment (*M* = 365.1 pg/mL, P5 = 156.2 pg/mL, P9 = 51,540.3 pg/mL) (*P* = 0.040). However, no significant change was detected in the non-responsive group before and after treatment. Similarly, no significant difference was observed in the MIC-1 between the response and nonresponse groups before treatment (*P* = 0.089), but a significant difference was detected after the treatment (*P* = 0.002) (Fig. 1).

### Distribution of MIC-1 genotype and allele frequency

**Fig. 2:**
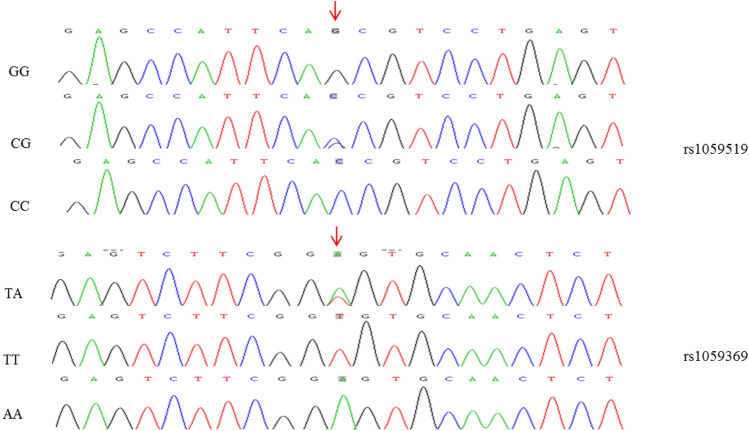
mic-1 polymorphism sequence. The arrow point indicates the corresponding SNP rs1059519[C/G] and rs1059369[A/T]

AA, AT, and TT genotypes were detected at rs1059369 of the *MIC-1* gene, and CC, CG, and GG genotypes were detected at rs1059519 locus (Fig. 2). The genotypes of rs1059519 and rs1059369 was in accordance with the law of genetic equilibrium by the Hardy–Weinberg equilibrium test. Chi-square test did not show any significant difference in the genotype and allele frequency of rs1059369 and rs1059519 between the responsive and non-responsive groups; a similar trend was observed among the SVR and non-SVR groups (*P* > 0.05). The linkage disequilibrium (LD) test showed an LD between rs1059369 and rs1059519 (*D*’ = 0.968, *r*^2^ = 0.275). Two SNPs of MIC-1 were constructed according to the results of LD. The haplotypes of A-C, A-G, and T-G were not significantly different between the responsive and non-responsive groups, and also between the SVR and non-SVR groups (*P* > 0.05) (Table 2).

**Table 2:** Genotype and allele distribution of different therapeutic groups in patients with CHC type 1b

Genotype	Evaluation of therapeutic effect at 12 weeks				Follow-up to 24 weeks for efficacy judgment			
	Response group (*n* = 124)	Nonresponse group (*n* = 47)	χ^2^, *P*	OR (95% CI)	SVR (*n* = 103)	Non-SVR (*n* = 69)	χ^2^, *P*	OR (95% CI)
rs1059369								
A	140 (0.565)	54 (0.574)	*χ*^*2*^ = 0.028	OR 1.04 (0.645–1.683)	114 (0.553)	80 (0.588)	*χ*^*2*^ = 0.405	OR 1.153 (0.744–1.787)
T	108 (0.435)	49 (0.426)	*P* = 0.868		92(0.447)	56 (0.412)	*P* = 0.525	
AA	40 (0.323)	16 (0.340)			31 (0.301)	25 (0.368)		
AT	60 (0.484)	22 (0.468)	*χ*^*2*^ = 0.052		52 (0.505)	30 (0.441)	*χ*^*2*^ = 0.904	
TT	24 (0.194)	9 (0.191)	*P* = 0.975		20 (0.194)	14 (0.191)	*P* = 0.636	
HWE	*χ*^*2*^ = 0.031	*χ*^*2*^ = 0.085			*χ*^*2*^ = 0.047	*χ*^*2*^ = 0.542		
	*P* = 0.860	*P* = 0.770			*P* = 0.828	*P* = 0.462		
rs1059519								
C	72 (0.290)	23 (0.245)	*χ*^*2*^ = 0.708	OR 0.79 (0.459–1.365)	56 (0.272)	39 (0.287)	*χ*^*2*^ = 0.091	OR 1.077 (0.665–1.744)
G	176 (0.710)	71 (0.755)	*P* = 0.400		150 (0.728)	97 (0.713)	*P* = 0.763	
CC	14 (0.113)	3 (0.064)			10 (0.097)	7 (0.1034)		
CG	44 (0.355)	17 (0.362)	*χ*^*2*^ = 0.942		36 (0.350)	25 (0.368)	*χ*^*2*^ = 0.095	
GG	66 (0.532)	27 (0.532)	*P* = 0.624		57 (0.553)	36 (0.529)	*P* = 0.954	
HWE	*χ*^*2*^ = 2.392	*χ*^*2*^ = 0.022			*χ*^*2*^ = 1.413	*χ*^*2*^ = 0.697		
	*P* = 0.122	*P* = 0.883			*P *= 0.235	*P* = 0.404		
haplotype								
A-C	70.70 (0.285)	23.00 (0.245)	*P* = 0.438	0.806 (0.467–1.391)	54.69 (0.265)	39.00 (0.287)	*P* = 0.692	1.103 (0680–1.789)
A-G	69.30 (0.279)	31.00 (0.330)	*P* = 0.376	1.260 (0.755–2.102)	59.31 (0.288)	41.00 (0.301)	*P* = 0.816	1.058 (0.658–1.701)
T-G	106.70 (0.430)	40.00 (0.426)	*P* = 0.907	0.972 (0.601–1.571)	90.69 (0.440)	56.00 (0.412)	*P* = 0.568	0.880 (0.567–1.365)
T-C	0.00 (0.000)	0.00 (0.000)			0.00 (0.000)	0.00 (0.000)		

### Comparison of MIC-1 level among different genotypes

**Fig. 3:**
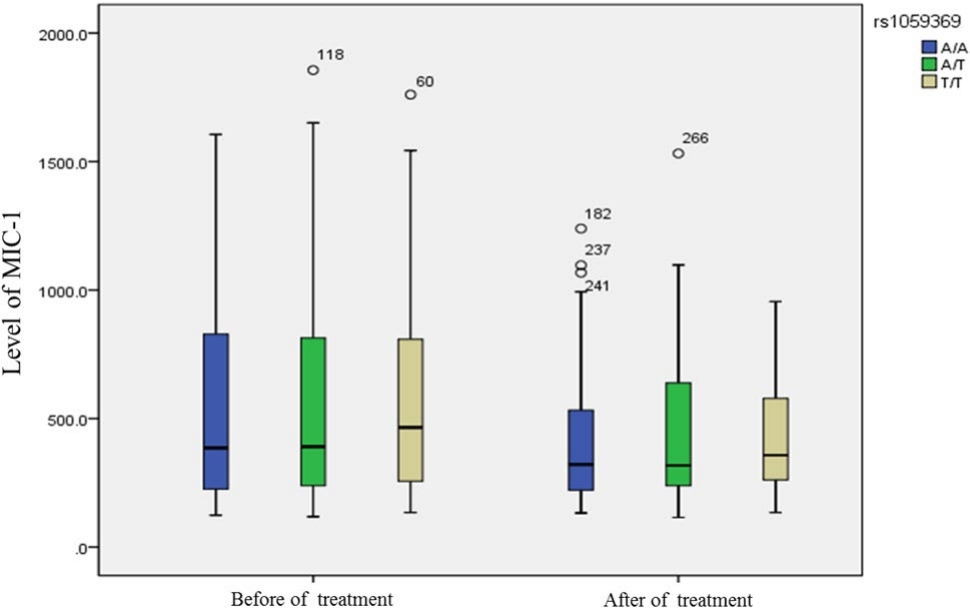
rs1059369 genotypes based levels of MIC-1 in CHC 1b patients before and after treatmentNo significant difference was detected in the MIC-1 level among different genotypes of rs1059369 in before or after treatment

**Fig. 4:**
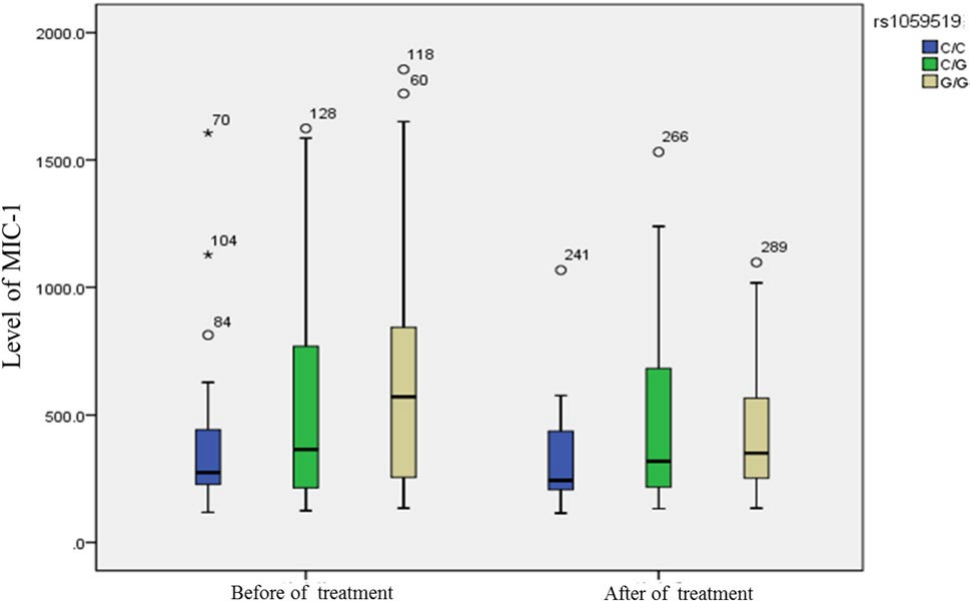
rs1059519 genotypes based levels of MIC-1 in CHC 1b patients before and after treatmentNo significant difference was detected in the MIC-1 level among different genotypes of rs1059519 in before or after treatment

The MIC-1 level of rs1059369 locus before treatment was as follows: AA (*M* = 385.60, P25 = 225.13, P75 = 834.25), AT (*M* = 390.65, P25 = 236.13, P75 = 814.70), and TT (*M* = 465.90, P25 = 254.55, P75 = 826.70). The MIC-1 levels after treatment were as follows: AA (*M* = 321.20, P25 = 221.30, P75 = 535.53), AT (*M* = 317.75, P25 = 236.53, P75 = 645.78), and TT (*M* = 357.60, P25 = 260.20, P75 = 582.25). No significant difference was detected in the MIC-1 level among different genotypes (*P* > 0.05). A similar result was observed in the MIC-1 levels at rs1059519 genotype before and after treatment (seen Figs. 3, 4). The findings are summarized in Table 2.

### Correlation between the level of MIC-1 and polymorphism and baseline parameters

After bivariate correlation analysis, the level of baseline MIC-1 was positively correlated with baseline ALT, AST, PIIINP, CIV, and *HCV* RNA in patients with type 1b CHC, negatively correlated with TP and ALB, but not correlated with TBIL, PLT, and age (*P* > 0.05).

Previous results showed that age and the levels of *ALT*, *PIIINP*, *c-IV*, and RNA of *HCV* were associated with efficacy; thus, 171 patients with CHC were divided into two groups according to these parameters. However, the genotype of rs1059369 and rs1059519 did not differ significantly with respect to age, PIIINP, CIV, and *HCV* RNA groups (*P* > 0.05). The results are shown in Table 3.

**Table 3:** Correlation between polymorphism of rs1059369 and rs1059519 and baseline parameters in patients with hepatitis C type 1b

Group	Case	rs1059369 Genotype			χ^2^	*P*	rs1059519 Genotype			χ^2^	*P*
		AA	AT	TT			CC	CG	GG		
Age (years)											
≤ 40	82	29 (0.354)	40 (0.486)	13 (0.159)	1.321	0.517	9 (0.110)	32 (0.390)	41 (0.500)	1.223	0.543
> 40	89	27 (0.303)	42 (0.472)	20 (0.225)			8 (0.09)	29 (0.326)	52 (0.584)		
ALT (U/L)											
≤ 50	92	32 (0.348)	46 (0.500)	14 (0.152)	2.144	0.342	8 (0.087)	33 (0.359)	51 (0.554)	0.353	0.838
> 50	79	24 (0.304)	36 (0.456)	19 (0.541)			9 (0.114)	28 (0.354)	42 (0.532)		
PIIINP (ng/mL)											
≤ 30	102	35 (0.343)	45 (0.441)	22 (0.216)	1.640	0.441	12 (0.118)	33 (0.324)	57 (0.559)	1.730	0.421
> 30	69	21 (0.304)	37 (0.536)	11 (0.159)			5 (0.072)	28 (0.406)	36 (0.522)		
CIV (ng/mL)											
≤ 30	96	33 (0.344)	45(0.469)	18 (0.188)	0.264	0.876	12 (0.125)	36 (0.375)	48 (0.500)	2.420	0.298
> 30	75	23 (0.307)	37 (0.493)	15 (0.200)			5 (0.067)	25 (0.333)	45 (600)		
HCV RNA											
≤ 2 × 10^6^	66	17 (0.258)	35 (0.530)	14 (0.212)	2.386	0.303	9 (0.136)	20 (0.303)	37 (0.561)	2.400	0.301
> 2 × 10^6^	105	39 (0.371)	47 (0.448)	19 (0.181)			8 (0.076)	41 (0.390)	56 (0.533)		

## Discussion

MIC-1 was first cloned from an activated macrophage cell line by Bootcov et al. [26]. MIC-1 is mainly involved in organ growth, differentiation, and development. In recent years, MIC-1 has been shown to be associated with chronic liver diseases, such as hepatitis and cirrhosis. Hsiao et al [27] demonstrated that MIC-1 levels increased significantly when the liver was damaged. Lee et al [28] reported a significant increase in serum MIC-1 levels in patients with cirrhosis and hepatocellular carcinoma, and the expression of MIC-1 in hepatocytes was significantly higher than that in adjacent para-cancerous and normal liver tissues. Yingyan et al [17] studied the expression of MIC-1 in patients with chronic hepatitis B and liver cirrhosis and found that the serum MIC-1 level was significantly increased in patients with chronic hepatitis B and liver cirrhosis, and the expression of MIC-1 in the liver tissues of patients with cirrhosis was significantly higher than that of patients with chronic hepatitis B. Si et al [29]showed that MIC-1 promotes HCV replication by altering the signal transduction and growth of host hepatocytes and is closely related to HCV-induced primary liver cancer. Jian et al [30] established a positive correlation between the level of MIC-1 in the serum of patients and CHC and left atrial diameter and left ventricular posterior wall thickness and a negative correlation with ejection fraction. Mohab et al [20] found that the level of MIC-1 in CHC patients was significantly higher than that in normal subjects.

The current study showed that the level of MIC-1 was positively correlated with ALT, AST, PIIINP, CIV, and *HCV* RNA in patients with CHC1b, suggesting that MIC-1 might be involved in HCV replication, liver cell injury, and liver fibrosis, and elevated plasma MIC-1 levels might be a potential diagnostic marker for HCV infection. However, only a few studies have reported a correlation between MIC-1 and the prognosis of CHC patients. Herein, we demonstrated that MIC-1 level in PR regimen response group decreased significantly after treatment, but no significant difference was detected in the MIC-1 level between pre-treatment and post-treatment in the nonresponse group, indicating that the altered plasma MIC-1 level is a marker of the efficacy of interferon. However, no significant difference was noted in the plasma MIC-1 levels between the responders and non-responders before treatment, suggesting that baseline MIC-1 levels in CHC patients were not significant predictors of interferon efficacy.

Importantly, the PR regimen can be applied to all patients with HCV genotype without contraindication, albeit the response rate is only about 60% [4]. Previous studies have shown that SNP in the host might have an impact on the antiviral therapy; for example, the CC genotype of rs12979860 of IL-28b, the TT genotype of rs809917, and the AA genotype of rsl2980275 was associated with spontaneous clearance of HCV infection and satisfactory response to PR regimen [31, 32]. Therefore, both the European guidelines for the diagnosis and treatment of HCV infection and the United States guidelines for the treatment of chronic HCV genotype 1 infection emphasize the use of IL-28b polymorphism to guide and predict the efficacy of PR. In addition, CD209 [33], IPTA [34], IL-10 [35], and other polymorphisms related to the treatment response of the PR regimen are also described. The rs1059369 locus of *MIC-1* gene harbors three genotypes: AA, AT, and TT. Another study showed that the polymorphism of rs1059369 is associated with the formation of collateral circulation in patients with non-ST-elevated myocardial infarction. Some other studies demonstrated that this locus does not affect MIC-1 protein expression and is not associated with cardiovascular disease and malignant tumor [36, 37]. The genotype of rs1059519 was CC, CG, and GG. Moreover, the genotype of rs1059519 was related to the susceptibility to chronic Keshan disease [38]. Chen et al [39] demonstrated the differences in the plasma MIC-1 levels between genotypes, which might be related to the severity of the disease as assessed by the plasma levels. Our previous study found the genotype and allele frequency distribution at the rs1059519 locus, not for rs1059369, significant differed between the patients and healthy controls. The genotype GG at the rs1059519 locus follow with high content of AST, PIIINP, MIC-1 were suggested as independent relevant factors for CHC [40]. In the current study, the genotype and allele frequency of the two sites did not differ significantly between the responsive and non-responsive groups and between the SVR and non-SVR groups (*P* > 0.05). Also, no significant correlation was established between the baseline clinical characteristics, plasma MIC-1 level, and SNPs (*P* > 0.05). Furthermore, the current study did not show any significant association between the polymorphism and the efficacy of PR regimens in patients with CHC 1b. Therefore, we believed that MIC-1 polymorphism would affect CHC chronic infection, while comprehensive results showed that MIC-1 polymorphism was of limited relevance to HCV treatment. Moreover, the polymorphism of both SNPs did not affect the level of MIC-1 expression in patients, which was inconsistent with that of the study by Chen et al. Thus, additional studies are required to confirm the findings.

## Conclusion

MIC-1 levels in the CHC patients with type 1b were correlated to liver cell injury, liver fibrosis index, and viral load, and the changes in the MIC-1 levels indicated the therapeutic effect of PR regimen, while the polymorphism of rs1059369 and rs1059519 did not affect the level of MIC-1 in CHC patients. Intriguingly, no significant correlation was established between the polymorphism of MIC-1 and the efficacy of antiviral therapy. The present study described the association of MIC-1 expression level to the efficacy of the PR regimen in CHC patients. However, the subjects were from Zhejiang Province, China, and the sample size was small, leading to selection bias in CHC patients. Thus, in future studies, the selection area and sample size should be expanded, and the influence of viral and environmental factors on HCV infection should be explored.
